# Dietary beer ameliorates glyoxylic acid-induced calcium oxalate nephrolithiasis in mice

**DOI:** 10.3389/fnut.2026.1963460

**Published:** 2026-10-19

**Authors:** Yaoyao Yang, Xinyu Deng, Junge Zhang, Yanping Dai, Xueqian Wu, Wei Liang

**Affiliations:** 1 School of Food Science and Engineering, Ningbo University, Ningbo, China; 2 Department of Clinical Laboratory, Xiangshan Red Cross Taiwan Compatriot Hospital Medical and Health Group, Xiangshan, Zhejiang, China; 3 Department of Laboratory Medicine, The First Affiliated Hospital of Ningbo University, Ningbo, China; 4 School of Laboratory Medicine and Life Science, Wenzhou Medical University, Wenzhou, China; 5 Zhejiang Engineering Research Center of Innovative Technologies and Diagnostic and Therapeutic Equipment for Urinary System Diseases, Ningbo, China

**Keywords:** beer, calcium oxalate, inflammation, nutritional epidemiology, oxidative stress, urolithiasis

## Abstract

**Introduction:**

Calcium oxalate (CaOx) nephrolithiasis is a major global health burden with limited non-pharmacological preventive options. Although moderate beer consumption has been linked to a lower risk of stone formation, the underlying mechanisms remain unclear. This study aimed to investigate the anti-lithogenic effects of dietary beer and the molecular pathways involved.

**Methods:**

We combined a retrospective clinical analysis of 415 urolithiasis patients with a glyoxylic acid–induced mouse model. Male C57BL/6J mice received low-/high-dose beer (3/6 mL/kg) by gavage. We assessed renal histology, urinary biochemistry, oxidative stress, inflammatory responses, and the expression of crystal-adhesion molecules (CD44, OPN, Kim-1).

**Results:**

In the clinical cohort, calcium oxalate monohydrate (COM) stones predominated (79.0%), with a male predominance (2.1:1). In mice, beer intervention lowered urinary oxalate and calcium concentrations, reduced histopathological injury and collagen deposition, downregulated CD44/OPN protein, decreased CD44/Kim-1/TNF-α/IL-1β mRNA expression, and partially preserved catalase and superoxide dismutase activities. The benefits were unlikely attributable to ethanol-induced diuresis, given the minimal gavage volume (~1.2–4% of fluid intake) and low ethanol delivery (~1.4–2.8 mg), which was further supported by unchanged daily water consumption.

**Discussion:**

These findings demonstrate that dietary beer attenuates CaOx stone formation and modulates urinary, inflammatory, oxidative, and adhesion markers. The anti-lithogenic potential of beer constituents supports the development of purine-free, non-alcoholic, hops-derived polyphenol preparations as a testable hypothesis. However, the absence of appropriate controls precludes attributing the effects to any single component, and these results should not be interpreted as a clinical recommendation to consume beer.

## 1 Introduction

Urinary tract stones are a common urological disease, with a rising prevalence globally, particularly in Asian regions. According to various epidemiological studies, the prevalence of urinary tract stones varies significantly across different regions and populations (1, 2). The formation of urinary tract stones is related to various factors, including genetics, dietary habits, lifestyle, and environmental factors (3). In recent years, with changes in dietary structure and lifestyle, the incidence of urinary tract stones has shown a rising trend (4, 5). Therefore, understanding the epidemiological characteristics and influencing factors of urinary tract stones is of great importance for the prevention and treatment of this condition (6, 7).

The etiopathogenesis of urolithiasis is inherently multifactorial, involving a complex interplay of genetic predisposition, environmental exposures, and metabolic derangements. Among these determinants, dietary habits have emerged as the most malleable and impactful modifiable risk factor, positioning nutritional intervention as a cornerstone of preventive nephrology (8, 9). While excessive alcohol intake is unequivocally associated with adverse metabolic consequences, including hyperuricosuria and dehydration, epidemiological evidence suggests a nuanced association with nephrolithiasis (10). Additionally, the polyuria caused by alcohol can dilute the concentration of various components in urine, which may be beneficial in preventing stone formation (11, 12). The putative protective effect of beer has conventionally been attributed to ethanol-mediated suppression of antidiuretic hormone (ADH) release from the neurohypophysis (13, 14), which increases free-water clearance and urine volume and thereby dilutes lithogenic solutes (15). This ADH-dependent diuretic hypothesis originates from human studies with large alcohol intake; diuresis is dose-dependent and not demonstrable at low rodent gavage doses (15, 16). Prospective evidence indicates each daily 240-mL beer serving associates with ~21% lower incident stone risk, persisting after adjustment for total fluid intake (17). Epidemiological evidence indicates that moderate to high consumption of beer and wine correlates with a lower incidence of nephrolithiasis (18).

Despite compelling observational correlations, the precise molecular mechanisms by which beer influences CaOx crystallization and renal tubular injury remain inadequately characterized. Prior studies have largely focused on isolated biochemical parameters, lacking integration with histopathological outcomes or the expression of crystal-adhesion molecules (19). To address this, the present study employed an integrative translational approach. We first conducted a comprehensive retrospective analysis of 415 urinary stone specimens to characterize the contemporary compositional and demographic distribution landscape of urolithiasis. Subsequently, utilizing a well-established glyoxylic acid-induced murine model of CaOx nephrolithiasis, we investigated the effects of standardized beer administration on renal function, urinary biochemistry, oxidative stress, inflammatory cascades, and the expression of key crystal-adhesion molecules.

## 2 Materials and methods

### 2.1 Materials

Glyoxylic acid solution was obtained from Aladdin (Catalog No. G169372-100 g). Beer (Snow, with an alcohol content of 2.9% and wort concentration of 8°P) was purchased from a local supermarket. Diethyl ether was obtained from the School of Food Science and Engineering, Ningbo University. The catalase (CAT) and superoxide dismutase (SOD) assay kits were purchased from the Jiancheng Bioengineering Institute (Nanjing, China). The HiPure Total RNA Mini Kit was purchased from Magen. The All-In-One 5X RT Master Mix Kit was purchased from abmart. The SuperStar Universal SYBR Master Mix Kit was purchased from CWBIO. Oxalate Assay Kit (Microplate Format) (Suzhou Grace Biotechnology Co., Ltd., Suzhou, China).

### 2.2 Methods

#### 2.2.1 Clinical sample collection

This retrospective study analyzed clinical data from 415 patients with urinary calculi treated at the First Affiliated Hospital of Ningbo University. Inclusion criteria were: (1) stone specimens obtained by surgery or extracorporeal shock-wave lithotripsy; and (2) complete clinical and pathological records. Exclusion criteria included insufficient specimen volume or missing key biochemical indicators. The cohort comprised 281 males and 134 females (mean age 55.0 ± 14.2 years). The protocol was approved by the Ethics Committee of the First Affiliated Hospital of Ningbo University (Approval No. 2024-156A), and all participants provided informed consent personally. This study analyzed the composition of submitted stone specimens. Due to the lack of a source-population denominator, it only describes the composition and age/sex distribution, and does not provide incidence data.

#### 2.2.2 Animal model

Male C57BL/6 J mice were purchased from GemPharmatech Co., Ltd. (Nanjing, China) and housed under specific-pathogen-free conditions at Ningbo University. Mice were randomly assigned to four groups (*n* = 10/group, Table 1) and given standard rodent chow and water ad libitum. After a 1-week acclimation with water, the experiment was conducted on study days 1–14 according to the schedule shown in Table 1. On days 1–7, beer (3 or 6 mL/kg) or an equal volume of water was administered by oral gavage once daily as a pre-treatment; on days 8–14, nephrolithiasis was induced by daily intraperitoneal injection of glyoxylic acid (100 mg/kg), and control mice received an equal volume of saline on the same schedule. Body weight was recorded daily; kidney weight was recorded post-mortem. Water was available ad libitum throughout; quantitative daily water-intake records and 24-h urine volumes were not obtained, and therefore 24-h solute excretion could not be calculated.

**Table 1:** Mouse grouping.

Group	Method
Control (*n* = 10)	Basal diet+saline
Model (*n* = 10)	Basal diet+100 mg/kg Glyoxylate induced
Model+low dose beer (*n* = 10)	3 mL/kg beer+100 mg/kg Glyoxylate induced
Model+high dose beer (*n* = 10)	6 mL/kg beer+100 mg/kg Glyoxylate induced

Dose selection and human equivalent dose (HED). Mice were gavaged with beer at 3 mL/kg (low) and 6 mL/kg (high), corresponding to ~60 and 120 μL for a 20 g mouse. These doses were selected to span a low-to-high exposure range within tolerable murine gavage volumes and to permit a dose–response assessment; the low dose was chosen near the minimal efficacious volume from pilot observations and the high dose as a two-fold increase to test linearity. They are not intended to model a specific human drinking pattern. At the beer’s 2.9% (v/v) ethanol content, this delivered ~1.37 and 2.75 mg ethanol per mouse per day (68.6 and 137.3 mg/kg). HED was calculated by the FDA body-surface-area method: HED = animal dose × (Km_mouse/Km_human), with Km = 3 for mouse and 37 for human, giving a divisor of 12.33 (20–22). The low and high doses thus correspond to ~14.6 and 29.2 mL beer/day for a 60 kg adult. These values only contextualize the administered dose and are not a dietary recommendation, as they do not account for interspecies differences in ethanol metabolism or constituent bioavailability.

At the end of the experiment, mice were anesthetized by inhalation of diethyl ether vapor. A piece of absorbent cotton soaked with 5–10 mL of diethyl ether was placed at the bottom of a sealed anesthesia chamber, where it volatilized to form an estimated diethyl ether vapor concentration of approximately 15–20% (v/v) in the chamber. Mice were placed inside the chamber, and once loss of the corneal reflex and muscle relaxation were observed (approximately 20–30 s), blood was immediately collected via eyeball enucleation, followed by euthanasia by cervical dislocation. We acknowledge that contemporary animal welfare guidelines increasingly favor volatile anesthetics such as isoflurane over diethyl ether; however, the diethyl ether anesthesia method used in this study involved only brief exposure. Future studies should adopt isoflurane induction anesthesia to align with current animal welfare standards.

All animal procedures were conducted in accordance with the guidelines and regulations approved by the Animal Ethics Committee of the Experimental Animal Center of Ningbo University and the National Research Council’s Guide for the Care and Use of Laboratory Animals (AEWC-NBU20250411). No animal died during the experiment and no animal or data point was excluded after randomization; all 40 randomized animals completed the study.

#### 2.2.3 Serum and urine collection and analysis

Mice were euthanized on day 15. Blood samples were collected and allowed to stand at room temperature for 2 h to facilitate clotting. Subsequently, the samples were centrifuged at 3,000 rpm for 10 min, and the supernatants (serum) were harvested. The collected serum was utilized to measure the levels of creatinine and blood urea nitrogen (BUN). Additionally, 24-h urine samples were collected from the mice. After storage at −80 °C, urine concentrations of oxalate (OA) were measured using a commercial assay kit strictly according to the manufacturer’s instructions, while calcium ion (Ca^2+^) levels were determined using a Beckman AU5800 automatic biochemical analyzer. Urine volume was not recorded; therefore all urinary results are reported as concentrations (mmol/L and mg/mL) and not as 24-h excretion, and urinary citrate, magnesium, uric acid and osmolality were not measured.

#### 2.2.4 Kidney histopathological analysis

Kidneys were dissected; the left and right kidneys were each divided into two parts. One portion was homogenized and stored in cryovials; the other was fixed in 4% paraformaldehyde and paraffin-embedded.

#### 2.2.5 HE staining

Paraffin-embedded kidney sections were stained with HE. To quantify CaOx crystal deposition, five non-overlapping fields per kidney section were randomly selected, and crystals were counted manually by two investigators blinded to group allocation. Crystal burden was further captured by the blinded composite renal histopathological injury score (Figure 1D; Table 2), whose criteria incorporate crystal number, aggregate size, and tubular obstruction. Morphological changes were evaluated across groups.

**Figure 1:**
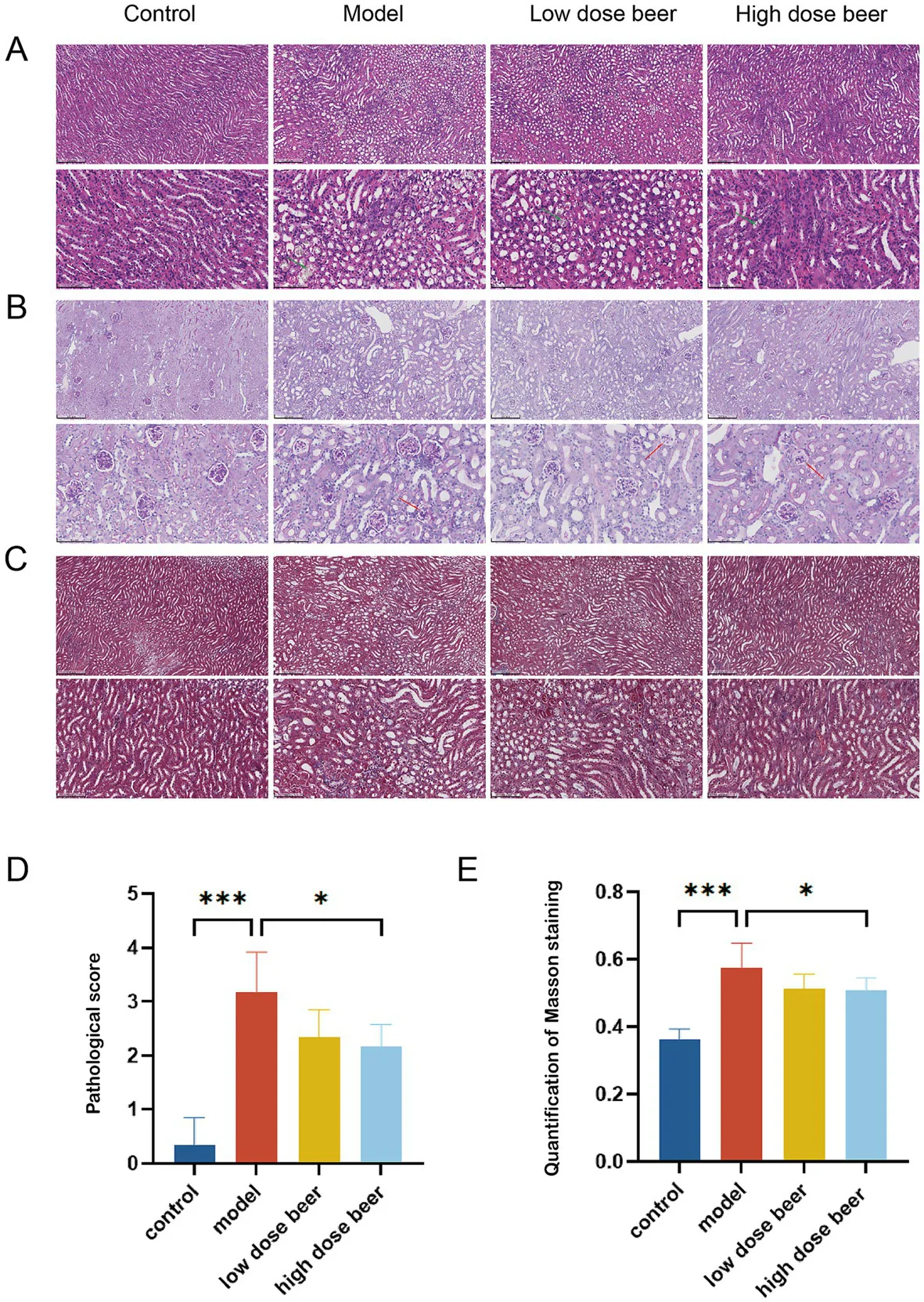
Beer mitigates renal tubular injury and fibrosis in nephrolithiasis mice. **(A–C)** Representative renal sections stained with HE **(A)**, PAS **(B)**, and Masson’s trichrome **(C)**; **(D,E)** Quantitative analysis of the composite renal histopathological injury score **(D)** and the Masson-positive collagen area fraction **(E)** (*n* = 6 per group; mean ± SD with individual values overlaid; scoring performed blinded to group). *p < 0.05, **p < 0.01, ***p < 0.001.

**Table 2:** Composite renal histopathological injury score.

Score	Degree of pathological change	Degree of injury
0	Intact glomeruli (open capillary loops, no atrophy); normal tubules (regular epithelium, continuous brush border, clear lumen); no crystals/casts; no inflammation/fibrosis	No Injury
1	No glomerular atrophy; slight tubular dilation, mild epithelial swelling, focal brush border loss; 1–3 scattered fine crystals (<10 μm, non-adherent)/field; occasional inflammatory cells	Mild
2	Focal glomerular atrophy (<10% glomeruli); moderate tubular dilation, epithelial flattening, extensive brush border loss; 4–8 crystals/field in small clumps (<50% lumen occupancy); focal inflammation	Moderate
3	Regional glomerular atrophy (10–30% glomeruli, capillary collapse); severe tubular dilation/collapse, widespread epithelial damage; 9–15 crystals/field fused into dense aggregates (≥50% lumen occupancy, partial obstruction); diffuse inflammation, mild fibrosis	Moderate-to-Severe
4	Diffuse glomerular atrophy (30–60% glomeruli, partial sclerosis); severe tubular disruption, extensive necrosis; >15 crystals/field causing complete tubular obstruction; dense inflammation, marked fibrosis/edema	Severe
5	Severe diffuse glomerular atrophy (>60% glomeruli, structural collapse); near-total tubular destruction, residual epithelial islands; near-total luminal occlusion by crystal masses; widespread fibrosis/edema, architecture effacement	Very Severe

#### 2.2.6 Masson staining

By applying Masson staining to observe the degree of renal fibrosis, collagen fibers are typically stained blue or green, muscle fibers appear red, and cell nuclei are stained dark blue or blackish-blue. Renal fibrosis was semi-quantitatively assessed by randomly selecting fields from Masson’s trichrome-stained sections to calculate the percentage of collagen deposition area. For each animal, three non-overlapping cortical fields were sampled from one section; the collagen-positive area was expressed as a fraction of the corresponding reference tissue area per field, and the three field values were averaged to yield a single value per animal, which was the unit of analysis.

#### 2.2.7 PAS staining

Paraffin sections were stained with PAS to visualize brush-border integrity, glycogen, and inflammatory infiltration within the glomeruli and tubules (23).

#### 2.2.8 Assessment of kidney functions and tubular necrosis score

To evaluate renal injury, kidney sections were stained with HE, PAS, and Masson’s trichrome to systematically assess pathological changes (e.g., renal crystallization, tubular dilatation, proteinaceous casts, interstitial fibrosis). Because no conventional scoring system captures the full spectrum of glyoxylate-induced injury in this model, a study-specific composite semi-quantitative score (0–5, Table 2) was used. This score integrates glomerular atrophy, tubular dilatation/epithelial damage, crystal number/luminal obstruction, inflammatory infiltration, and interstitial fibrosis/oedema, with criteria adapted from previously described grading systems for glyoxylate-induced renal injury (24, 25).

#### 2.2.9 Immunohistochemistry

The expression and localization of CD44 and OPN proteins in tissues were detected using immunohistochemical staining. The main experimental steps include: routine deparaffinization of paraffin sections, rehydration through graded ethanol, and antigen retrieval, followed by sequential incubation with specific primary antibodies against CD44 and OPN and corresponding secondary antibodies, visualization with DAB, and finally counterstaining with hematoxylin, dehydration, clearing, and mounting.

Staining results were observed using a scanning software. Semi-quantitative analysis of stained images was performed using professional image analysis software (ImageJ). DAB-positive signals were segmented using a uniform color threshold. The software output the positive area (Area), integrated optical density (IOD), and the percentage of positive area (%Area). The primary endpoint was mean optical density (MOD = IOD/Area). For each animal, one section was taken, and three non-overlapping cortical fields were selected by systematic random sampling; values from each field were averaged to yield a single representative value per animal, with the animal as the statistical unit. All measurements were performed by a single operator blinded to group allocation.

#### 2.2.10 Oxidative stress markers

Renal tissue was homogenized in physiological saline at 1:9 (w/v) on ice and centrifuged at 4,000 rpm for 10 min at 4 degrees C. Supernatants were used to measure protein concentration; CAT and SOD activities were determined by kit at 405 nm and 450 nm, respectively, per the manufacturer’s instructions (26, 27).

#### 2.2.11 Inflammatory cytokines

Renal TNF-α and IL-1β expression was evaluated at the mRNA level by quantitative real-time PCR.

#### 2.2.12 Expression of Kim-1 and CD44 in renal tissues

In the present study, immunohistochemical (IHC) staining was performed to assess the protein expression levels of CD44 and osteopontin (OPN), while quantitative real-time PCR (qPCR) was employed to further determine the mRNA expression levels of Kim-1 and CD44.

#### 2.2.13 RNA extraction and quantitative real-time PCR

According to the manufacturer’s instructions, total RNA was extracted using the HiPure Total RNA Mini Kit. The All-In-One 5X RT Master Mix (Abmart) was used to reverse-transcribe 1–2 μg of RNA. Real-time PCR was performed on a thermal cycler using the SuperStar Universal SYBR Master Mix (CWBIO, China). Primers used are as shown in Table 3.

**Table 3:** DNA sequences of primers for polymerase chain reaction.

Gene name	Primer name	Sequence (5′-3′)
GAPDH	GAPDH-F	TGAACGGGAAGCTCACTGG
	GAPDH-R	TCCACCACCCTGTTGCTGTA
TNF-α	TNF-α-F	ATGTCTCAGCCTCTTCTCATTC
	TNF-α-R	GCTTGTCACTCGAATTTTGAGA
IL-1β	IL-1β-F	AGCAGCTATGGCAACTGTTC
	IL-1β-R	AATGAGTGATACTGCCTGCC
CD44	CD44-F	TGGATCCGAATTAGCTGGACA
	CD44-R	TGCAGACGGCAAGAATCAGA
Kim-1	Kim-1-F	GAGAGTGACAGGGTCTGTATTG
	Kim-1-R	CCTTGTAGTTGTGGGTCTTCTT

#### 2.2.14 Statistical analysis

Data are presented as mean ± SD. In this study, inflammatory, biochemical, and renal CAT/SOD activities were prospectively defined as the co-primary endpoints, with 10 mice enrolled per group. All remaining endpoints, including histological and other measures, were recognized as secondary/exploratory, and no formal multiplicity adjustment was performed. Prior to parametric testing, normality (Shapiro–Wilk) and homogeneity of variance (Bartlett’s test) were verified. Body-weight trajectories (Figure 2A) were analyzed by two-way repeated-measures ANOVA (time x group) with post-hoc comparison at each time point; other endpoints were analyzed by one-way ANOVA followed by Tukey’s post-hoc test (GraphPad Prism 9.5). Histological scoring and image quantifications were performed blinded; multiple fields per animal were averaged to a single value, so *n* denotes the number of animals. *p* < 0.05 was significant.

**Figure 2:**
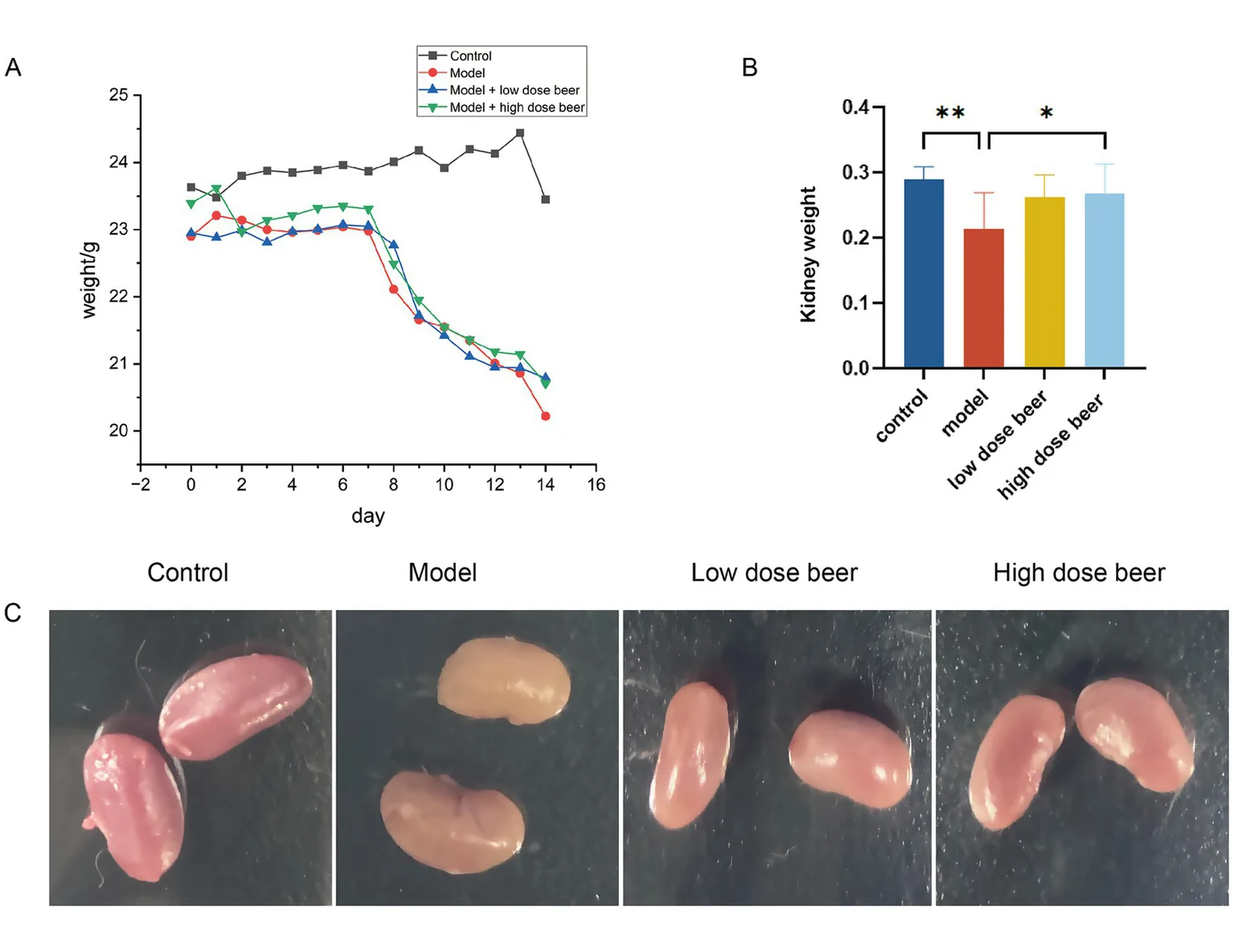
Beer attenuates body weight loss and ameliorates renal atrophy in the mouse model. **(A)** Body weight curves of mice throughout the 14-day experimental period (*n* = 10 per group; mean ± SD). **(B)** Absolute wet kidney weight of mice at the end of the experiment (*n* = 10 per group; mean ± SD). **(C)** Representative gross photographs of excised kidneys from each experimental group. *p < 0.05, **p < 0.01.

## 3 Results

### 3.1 Demographic and compositional characteristics of clinical samples

A total of 415 stone specimens were included in this study. Qualitative and quantitative analyses of stone composition revealed that the detected components were predominantly calcium oxalate-based (Table 4). Specifically, calcium oxalate monohydrate (COM) had the highest detection rate at 79.0% (328/415), followed by carbonate apatite (CA) at 54.9% (228/415), and calcium oxalate dihydrate (COD) at 41.0% (170/415). The detection rate of uric acid (UA) was 9.2% (38/415). Other less common components included brushite (6.0%), struvite (4.3%), protein (4.1%), and cystine (0.2%). Notably, seven specimens contained “sodium hydrosulfide hydrate; sodium hydroxide,” which may be attributed to sample processing artifacts or environmental contaminants. In terms of demographics, male patients significantly outnumbered females, with a male-to-female ratio of 2.1:1 (males: 281 cases, 67.7%; females: 134 cases, 32.3%).

**Table 4:** Stone composition profile.

Component	Male	Female	Number of cases	Percentage (%)
Calcium Oxalate Monohydrate	237	91	328	79.0
Carbonate Apatite	134	94	228	54.9
Calcium Oxalate Dihydrate	114	56	170	41.0
Uric Acid	31	7	38	9.2
Brushite	12	13	25	6.0
Struvite	11	7	18	4.3
Protein	7	10	17	4.1
Sodium Hydrosulfide Hydrate; Sodium Hydroxide	6	1	7	1.7
Ammonium Urate	3	1	4	1.0
Cystine	0	1	1	0.2

Age-stratified analysis showed that calcium oxalate monohydrate (COM; 39.2%), carbonate apatite (CA; 27.3%), and calcium oxalate dihydrate (COD; 20.3%) were the most prevalent components, accounting for 86.8% of all cases. Patients with uric acid stones (including uric acid and ammonium urate) were significantly older (mean ages 61.9 and 62.2 years, respectively) compared to those with calcium-based stones (mean ages ranging from 52.4 to 55.0 years). Other less common components, such as brushite, struvite, protein, and cystine, together constituted the remaining 8.6% of cases (Table 5).

**Table 5:** Stone composition by age group and mean patient age.

Component	<30	30–39	40–49	50–59	60–69	70	Mean age	SD of age
Calcium Oxalate Monohydrate	8	41	51	103	81	44	55	13
Carbonate Apatite	8	37	39	77	38	29	52.7	13.5
Calcium Oxalate Dihydrate	5	29	27	59	32	18	52.4	12.8
Uric Acid	0	1	4	13	10	10	61.9	10
Brushite	1	1	5	7	6	5	56.4	14.8
Struvite	0	4	4	2	2	6	54.9	16.1
Protein	1	1	0	7	6	2	57.6	12.8
Sodium Hydrosulfide Hydrate; Sodium Hydroxide	1	0	0	4	2	0	51	13.4
Ammonium Urate	0	0	1	1	0	2	62.2	14.6
Cystine	0	0	0	1	0	0	51	/

In view of the clinical observations that calcium oxalate monohydrate (COM) constituted the majority of stones (79.0%), that mixed compositions were prevalent (83.9%), and that a significant male bias existed within the cohort, a murine model of calcium oxalate urolithiasis was established using glyoxylate induction in male subjects. The study design included a control group, a model group, and a dietary intervention group.

### 3.2 Beer ameliorates body weight loss and renal atrophy in mice

Control mice gained weight steadily over the 14-day period (Figure 2A), whereas the model group showed a significant decline from approximately day 6 onward (*p* < 0.01 vs. control). Low- and high-dose beer attenuated this reduction (*p* < 0.05 and *p* < 0.01, respectively). Kidney wet weight was significantly lower in the model than the control group (*p* < 0.01, Figure 2B), and beer, especially at the high dose, restored it toward control levels. (*p* < 0.05), reflecting improved renal morphology. Gross examination (Figure 2C) showed normal reddish, smooth kidneys in controls, whereas model kidneys were pale, shrunken, and atrophic.

To exclude an ethanol-diuresis dilution mechanism, daily water intake was recorded (Table 6). Controls remained stable (~3.6 mL/animal/day); model and beer groups showed progressive reduction during induction, with no polydipsia, consistent with glyoxylate-induced anorexia. Thus, lower urinary oxalate/calcium from beer is not attributed to increased fluid intake, arguing against ethanol-driven dilution; however, 24-h urine volume and osmolality were not measured, so a dilution effect cannot be fully excluded.

**Table 6:** Daily water consumption (mL/animal/day).

Day	Control	Model	Low dose beer	High dose beer
1	3.36	3.8	3.46	3
2	4	4	4	3.44
3	3.8	3.86	4	4.56
4	3.58	3.74	3.74	3.9
5	3.96	3.82	3.72	3.8
6	3.84	3.98	3.44	3.8
7	3.84	4.12	3.9	4.28
8	3.66	4	3.78	4.34
9	2.86	3.2	2.24	2.12
10	3.14	3	1.92	1.64
11	3.94	3.3	2.42	2.4
12	3.76	2.06	1.64	2
13	3.52	2.46	1.86	2.2
14	3.34	2.2	2	1.8

### 3.3 Beer-mediated renoprotection and urinary metabolic regulation in mice

Deposition of calcium oxalate crystals induced acute tubular injury, acute or chronic interstitial nephritis, and elevated serum creatinine levels (28). Compared with controls, the model group exhibited significantly elevated serum creatinine (Cr; Figure 3A) and blood urea nitrogen (BUN; Figure 3B), indicating severe renal impairment. Both low- and high-dose beer treatments markedly reduced these levels, demonstrating a renoprotective effect. Furthermore, beer alleviated the marked increases in urinary oxalate (OA; Figure 3C) and calcium (Ca^2+^; Figure 3D) observed in the model group.

**Figure 3:**
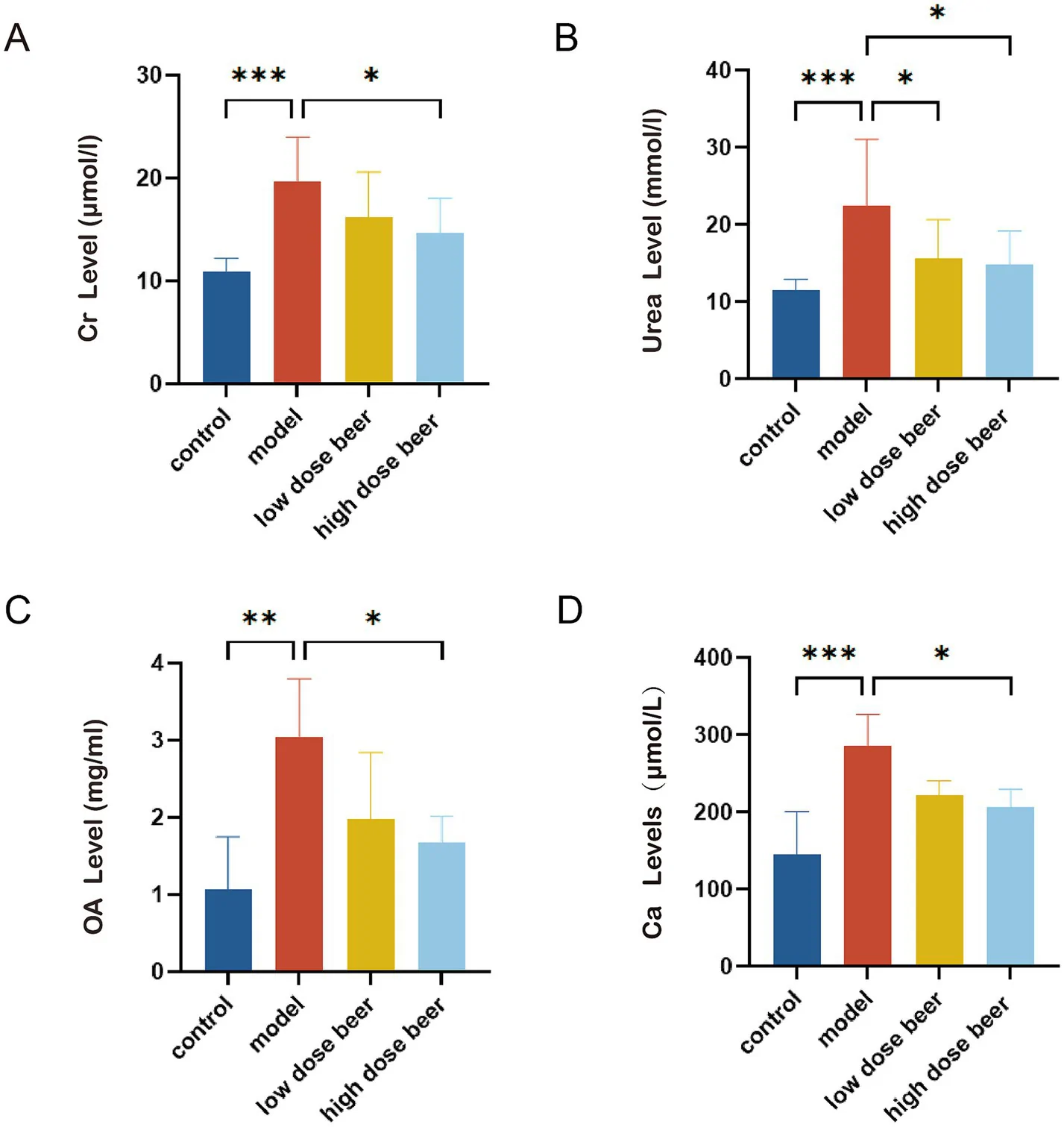
Effects of beer administration on renal function and urinary compositions in lithogenic mice. **(A)** Serum creatinine (Cr) levels. **(B)** Blood urea nitrogen (BUN) levels. **(C)** Urinary oxalate (OA) concentrations. **(D)** Urinary calcium (Ca^2+^) concentrations (*n* = 10 per group; mean ± SD with individual values overlaid). *p < 0.05, **p < 0.01, ***p < 0.001.

### 3.4 Beer alleviates glyoxylate-induced renal pathological injury

To evaluate the effects of beer on renal histomorphology, we performed H&E, PAS and Masson’s trichrome staining on kidney sections from all groups. HE staining (Figure 1A) showed that the control group had intact renal architecture with no congestion or interstitial inflammatory infiltration, the model group showed significant impairment of renal function and pronounced crystal deposits upon histological staining. Compared with the model group, the beer groups showed a reduction in the number of crystals.

Cell apoptosis is associated with CaOx crystal formation and renal injury (29). PAS staining further elucidated the mitigating effect of beer on renal tubular epithelial injury. The control group exhibited intact renal tubules and glomeruli, characterized by a continuous and dense brush border (Figure 1B). In contrast, the model group displayed marked tubular dilation and glomerular atrophy, accompanied by abundant PAS-positive cast deposition within the lumen, indicating significant proximal tubular damage. Although beer intervention reduced the accumulation of PAS-positive materials in the tubular lumen, glomerular atrophy persisted in the treatment groups.

Tubulointerstitial fibrosis has been reported as a key pathophysiological process in oxalate nephropathy with renal failure. To evaluate the alterations of fibrosis levels in glyoxylate-damaged renal tissue in four groups, we performed Masson staining. Masson’s trichrome staining revealed extensive collagen deposition in the renal interstitium of the model group, indicative of severe tubulointerstitial fibrosis (Figure 1C). Notably, beer intervention reduced this collagen fiber accumulation. Quantitative analysis (Figure 1E) confirmed that the Masson-positive area was substantially elevated in the model group compared to the control group, whereas it decreased significantly in the beer groups. These findings indicate that beer effectively mitigates the progression of renal fibrosis induced by lithogenic stimuli.

### 3.5 Beer alleviates glyoxylate-induced renal injury and crystal deposition

Negatively charged protein molecules, such as CD44 and osteopontin (OPN), often serve as nucleation sites for crystal growth (30). These molecules facilitate crystal growth, aggregation, and enlargement, thereby significantly increasing the propensity for crystal retention within the kidney (1, 31). Immunohistochemical staining showed strong CD44 and OPN expression in the renal tubular epithelium of the model group, appearing as dense brown precipitates across tubular regions; quantitative analysis confirmed a higher positive area for both proteins versus controls. Beer intervention was associated with attenuated expression of these two proteins, as shown by paler, less extensive staining in both beer groups, suggesting that beer reduces the availability of crystal adhesion factors. Whether this reflects a direct action of a specific beer constituent or an indirect consequence of reduced crystal load remains to be determined.

In mouse models of urolithiasis, Kim-1 functions not merely as a straightforward injury indicator (32), but also serves a crucial role in early diagnosis and the pathological clearance of damaged cells. qPCR showed significantly upregulated CD44 and Kim-1 mRNA in model mice versus controls (Figures 4E,F), and beer intervention - particularly at the high dose - markedly attenuated this upregulation. These data are consistent with beer lessening tubular epithelial stress, although they do not by themselves establish a causal pathway.

**Figure 4:**
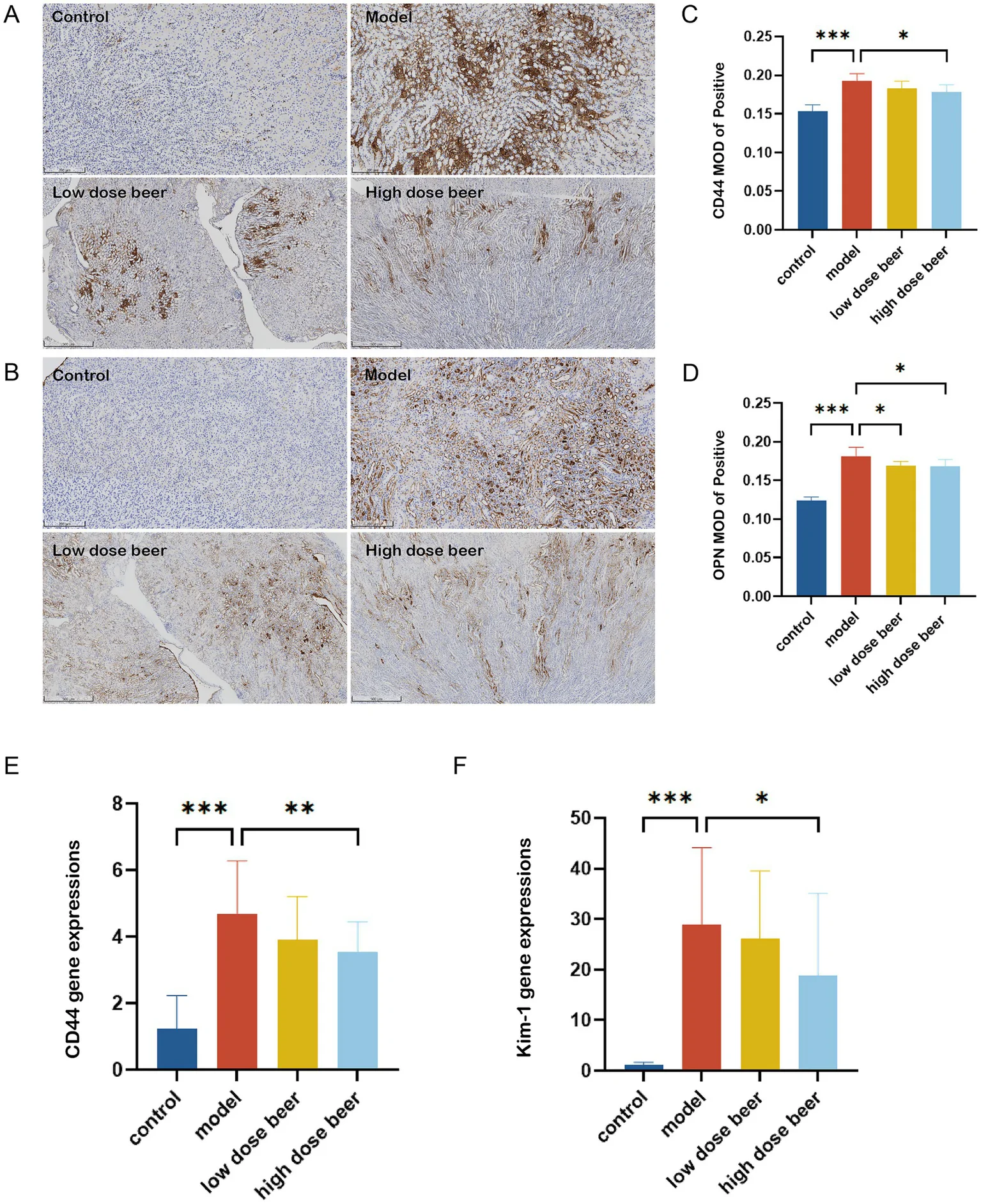
Beer attenuates renal tubular injury by downregulating CD44, OPN, and Kim-1 expression. **(A,B)** Representative immunohistochemical (IHC) staining images showing the expression of CD44 **(A)** and Osteopontin (OPN) **(B)** in mouse renal tissue sections; **(C,D)** Quantitative analysis of the mean optical density of the positive signal (MOD = IOD/positive area) for CD44 **(C)** and OPN **(D)**; **(E,F)** Relative mRNA expression levels of CD44 **(E)** and Kidney Injury Molecule-1 (Kim-1) **(F)** determined by quantitative real-time PCR (qPCR) (*n* = 6 per group for histology; *n* = 10 per group for qPCR; mean ± SD with individual values overlaid; quantification performed blinded to group). *p < 0.05, **p < 0.01, ***p < 0.001.

### 3.6 Beer alleviates glyoxylate-induced renal oxidative stress and inflammation

Oxidative stress and inflammation play pivotal roles in the pathogenesis of renal injury (33). CaOx stone formation is closely linked to inflammation and oxidative stress (34, 35). The model group exhibited significant upregulation of pro-inflammatory cytokines TNF-α and IL-1β mRNA and markedly reduced activities of antioxidant enzymes CAT and SOD. Beer intervention partially reversed these changes, with decreased cytokine expression and partial preservation of enzyme activities, reflecting attenuation of glyoxylate-induced activity loss rather than normalization. Specifically, only the high-dose beer group showed a statistically significant reduction in TNF-α and significant recovery of CAT and SOD activities; changes in IL-1β did not reach statistical significance. These findings indicate that beer attenuates glyoxylate-induced renal inflammation and partially preserves enzymatic antioxidant capacity (Figure 5).

**Figure 5:**
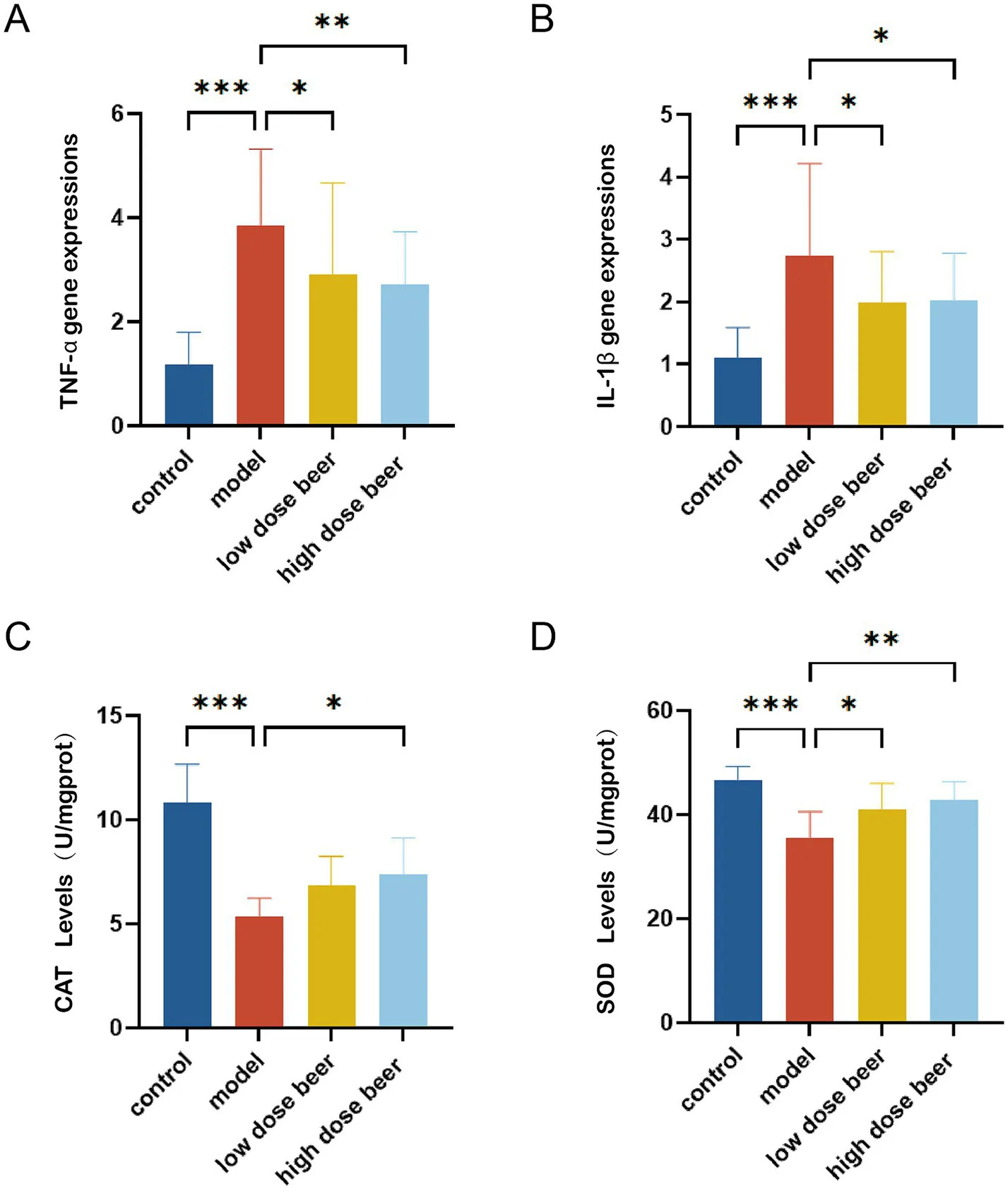
Representative data on renal inflammation and oxidative stress markers. **(A,B)** Relative mRNA expression levels of TNF-α **(A)** and IL-1β **(B)** in renal tissues. **(C,D)** Enzymatic activities of catalase (CAT) **(C)** and superoxide dismutase (SOD) **(D)** in renal homogenates (*n* = 10 per group; mean ± SD with individual values overlaid). *p < 0.05, **p < 0.01, ***p < 0.001.

## 4 Discussion

Calcium oxalate (CaOx) nephrolithiasis represents a significant global health burden, contributing substantially to chronic kidney disease morbidity and imposing considerable economic costs on healthcare systems. While current clinical management focuses on surgical intervention and pharmacological agents to prevent recurrence, patient compliance often remains suboptimal due to side effects. Consequently, identifying modifiable dietary factors that mitigate lithogenic risk has become a research priority in nutritional sciences (36, 37). In this study we integrated a descriptive clinical case series analysis of 415 stone specimens with a glyoxylic acid-induced murine model to characterize the effects of beer on CaOx nephrolithiasis. Our results show that, at the doses tested, beer administration was associated with attenuated CaOx stone formation, concurrent with a multi-target shift in urinary biochemistry, crystal-adhesion molecule expression, inflammation, and antioxidant enzyme activities. We emphasize, however, that these endpoints are correlations; the study does not establish that any specific pathway mediates the effect, and the active constituent(s) remain unidentified.

Clinically, our case series data show that CaOx - particularly the monohydrate form (COM, 79.0%) - is the predominant stone constituent in this Chinese cohort, with a marked male predominance (2.1:1). These patterns align with large registries and justify focusing on CaOx pathogenesis. The demographic skew likely reflects sex-specific metabolic differences, including androgen-driven hepatic oxalate synthesis and lower urinary citrate in males versus premenopausal females (38). Importantly, this clinical component is descriptive: it characterizes stone composition and demographics but did not record beer consumption, so it provides background context for the disease rather than evidence for or against a protective effect of beer. It also lacks a denominator for the source population, so the age and sex patterns reported here describe the distribution of the included cases and cannot be interpreted as incidence or as evidence of higher risk in men.

A critical insight from our study lies in the regulation of urinary biochemistry. Hyperoxaluria and hypercalciuria are the primary physicochemical drivers of CaOx supersaturation and precipitation. We observed that beer intervention reduced urinary concentrations of both oxalate and calcium (18). In the epidemiological literature, this reduction is conventionally attributed to ethanol’s diuretic effect, which suppresses antidiuretic hormone (ADH), increases urine flow, and dilutes lithogenic solutes. In the present experiment, however, such a physical dilution mechanism is unlikely. The gavaged volumes were only ~60 μL (low) and 120 μL (high) per 20 g mouse—roughly 1.2–4% of the reported daily fluid intake of C57BL/6 J mice (3–5 mL/day)—and the accompanying ethanol load was correspondingly minute (~1.37 and 2.75 mg/mouse/day, i.e., 68.6 and 137.3 mg/kg). A burden of this magnitude would not produce a physiologically meaningful diuresis nor suppress ADH sufficiently to alter systemic solute concentrations. However, this argument rests on concentrations rather than on 24-h excretion. Because we did not measure 24-h urine volume, urine osmolality, or inhibitory factors, we cannot distinguish whether the observed changes are due to reduced solute excretion, a diuretic effect, or dilution. We therefore present only a hypothesis: the reduction in urinary oxalate and calcium is driven by trace beer constituents modulating intestinal/renal tubular function, rather than by dilution. Future studies require concurrent 24-h urine volume measurement, isovolumetric water-loaded, non-alcoholic beer, and ethanol-matched control groups, along with targeted metabolomic profiling.

Beyond physicochemical modulation, our results highlight the pivotal role of the renal tubular epithelial microenvironment in stone retention. Studies have shown that persistent expression of Kim-1 in renal tubular epithelial cells of Kim-1-overexpressing transgenic mice contributes to the development of renal fibrosis (32). OPN and CD44 play an important role in the adhesion of renal epithelial cells to CaOx crystals (39). CaOx crystal adhesion to renal tubular cells, mediated by glycoproteins (e.g., CD44, OPN), is a prerequisite for stone maturation. Glyoxylic acid upregulated CD44/OPN to promote crystal binding, whereas beer downregulated these adhesion molecules. Given that beer hops are rich in polyphenols, we hypothesize that their anti-adhesive properties contribute to reduced crystal retention. However, limitations exist: the beer was not chemically characterized, and changes may reflect reduced crystal burden and secondary injury rather than direct polyphenol action; moreover, the lack of ethanol-matched and non-alcoholic beer controls precludes distinguishing ethanol from polyphenol contributions. Future studies should isolate specific hops polyphenols, clarify their mechanisms on adhesion molecules and nucleation, and establish a basis for non-alcoholic, ethanol-independent prevention strategies.

From the perspective of antioxidant specificity, the present results should be distinguished from the renoprotective effects reported for other dietary polyphenols. Resveratrol, for example, acts largely through SIRT1/AMPK activation and direct reactive-oxygen-species scavenging (36). Hops-derived polyphenols share these redox-modulating properties, yet our data point to an additional, stone-specific axis: by downregulating CD44, OPN, and Kim-1 they interfere directly with crystal–cell adhesion and the early nucleation environment, an effect not typically ascribed to resveratrol. This specificity, however, remains putative, because the present design cannot isolate hops polyphenols from the other constituents of whole beer; component-resolved studies are required to confirm it.

Furthermore, we elucidated the interplay between crystal deposition and maladaptive tissue remodeling. The accumulation of CaOx crystals triggers oxidative stress and sterile inflammation, leading to tubular necrosis and interstitial fibrosis (25). Model data fit this paradigm: elevated TNF-α/IL-1β and reduced CAT/SOD, interpreted as enzymatic antioxidant depletion under oxidative insult. Beer partially restored these activities, indicating lower oxidant burden. However, CAT/SOD measure antioxidant capacity, not oxidative damage itself; without direct markers, evidence for oxidative stress is indirect, and suppressed (vs. exhausted) antioxidant response cannot be excluded.

From a nutritional epidemiology standpoint, these mechanistic findings provide biological plausibility to previous observational studies (18). However, translating these findings into clinical or public-health recommendations requires explicit recognition of a genuine clinical paradox. Beer carries a high purine load that metabolizes to uric acid, increasing uric acid concentrations, lowering urinary pH, and fostering uric acid crystallization and heterogeneous nucleation of calcium oxalate. The net trade-off is therefore uncertain: any benefit against CaOx stones must be weighed against a raised risk of uric acid stones—a concern directly pertinent to our cohort, where uric acid was found in 9.2% (38/415) of specimens. Combined with the established hazards of excessive alcohol intake, this explains why clinicians do not recommend beer for CaOx-stone prevention, and our findings must not be read as an endorsement of beer drinking. The translational aim is not to promote beer as a beverage but to “deconstruct” it—isolating, purifying, and standardizing the anti-lithogenic hops-derived polyphenol fraction into a purine-free, non-alcoholic formulation—so that patients could, in principle, gain the observed benefits while avoiding the risks of ethanol and purine. This will require component-resolved studies directly comparing whole beer, non-alcoholic beer, an ethanol-matched control, and purified hops-polyphenol fractions.

Several limitations should be noted: (1) The retrospective single-centre clinical cohort may have selection bias and lacks a source-population denominator, precluding incidence/risk estimation. (2) The glyoxylate mouse model mimics acute CaOx crystallization but not chronic human stone disease. (3) Absence of ethanol-matched/non-alcoholic beer controls prevents distinguishing ethanol, polyphenols, and other constituents; uncharacterized beer limits mechanistic claims to hypothesis. (4) Human-equivalent doses are body-surface-area estimates ignoring interspecies metabolic differences. (5) Lack of urine volume, osmolality, water intake, and uric acid data precludes diuretic/purine-load assessment and 24-h excretion calculation. (6) Small sample sizes for imaging endpoints limited statistical power, rendering them exploratory; conclusions rely mainly on biochemical/molecular endpoints.

## 5 Conclusion

This study combined a descriptive clinical case series and a glyoxylate-induced mouse model to elucidate the regulatory effects of beer administration on CaOx kidney stones, providing novel evidence for dietary nutrient involvement in urolithiasis. Clinically, calcium oxalate monohydrate (COM) predominated in the Chinese population (79.0%), and men were more frequently represented than women (2.1:1); as a descriptive series without a denominator, these data describe the composition and case distribution of included stone specimens and do not establish incidence or a higher risk in men. Experimentally, beer administration conferred renoprotection via multi-target mechanisms: (1) associated with reduced urinary oxalate and calcium concentrations, consistent with altered CaOx supersaturation; because 24-h urine volume was not measured, these concentration data do not establish a solute-specific rather than dilution-based mechanism. (2) correlated with downregulated crystal-adhesion molecules (CD44, OPN, Kim-1), implying reduced crystal retention; (3) accompanied by lower levels of pro-inflammatory cytokines (TNF-α, IL-1β); and (4) paralleled by partial preservation of the antioxidant enzyme (CAT, SOD) activities that were reduced by glyoxylic acid, coinciding with attenuated oxidative stress and renal interstitial fibrosis.

Crucially, these findings do not endorse beer as a prophylactic against kidney stones. Beer delivers a substantial purine load metabolized to uric acid, raising uric acid-stone risk—a concern underscored by uric acid in 9.2% of our cohort specimens—and excessive alcohol carries established hazards (hepatotoxicity, hypertension, addiction). Clinicians should therefore not recommend beer for stone prevention. The translational aim is to isolate, purify, and standardize the hops-derived polyphenol fraction into a purine-free, non-alcoholic preparation, delivering the putative anti-lithogenic benefit without the associated alcohol and purine risks. Key limitations include the absence of an ethanol-matched control and a non-alcoholic beer arm, which precludes attribution to any single constituent; selection bias from the single-centre, retrospective design; the murine model’s incomplete recapitulation of human idiopathic stone disease; and that the reported human equivalent dose (~14.6–29.2 mL/day for a 60 kg adult) is a body-surface-area estimate that ignores interspecies differences in ethanol or polyphenol handling. The hops-polyphenol mechanism advanced here is a hypothesis: the beer was not chemically characterized and the observed changes in CD44, OPN, Kim-1, inflammatory markers and antioxidant enzymes may reflect a lower crystal burden and reduced renal injury rather than a direct action of hops polyphenols. Future studies should prioritize component-resolved comparisons of whole beer, non-alcoholic beer, ethanol-matched controls, and purified hops-polyphenol fractions.

## Data Availability

The original contributions presented in the study are included in the article/supplementary material, further inquiries can be directed to the corresponding author.
